# Supplementary material for: Chemical and Biological Evaluation of Amazonian Medicinal Plant Vouacapoua americana Aubl
Source: Plants (Basel). 2022 Dec 25;12(1):99. doi: 10.3390/plants12010099 (PMC9824835; doi:10.3390/plants12010099)

Supporting Information

Chemical and Biological Evaluation of Amazonian Medicinal Plant *Vouacapoua americana* Aubl.

Serhat Sezai Çiçek [1,*], Anna Laís Pfeifer Barbosa [1], Arlette Wenzel-Storjohann [2], Jorge Federico Orellana Segovia [3], Roberto Messias Bezerra [4], Frank Sönnichsen [5], Christian Zidorn [1], Isamu Kanzaki [6] and Deniz Tasdemir [2,7]

1 Department of Pharmaceutical Biology, Kiel University, Gutenbergstr. 76, 24118 Kiel, Germany

2 Research Unit Marine Natural Products Chemistry, GEOMAR Centre for Marine Biotechnology (GEOMAR-Biotech), GEOMAR Helmholtz Centre for Ocean Research Kiel, Am Kiel-Kanal 44, 24106 Kiel, Germany

3 Ecoregional Research Unit, Brazilian Agricultural Research Corporation, Rod. JK, Km 5, Macapá, 68903-419 Amapá, Brazil

4 Laboratory of Bioprospection and Atomic Absorption, Federal University of Amapá, Rod. JK, Macapá, 68903-419 Amapá, Brazil

5 Otto Diels Institute for Organic Chemistry, Kiel University, Otto-Hahn-Platz 4, 24118 Kiel, Germany

6 Laboratory of Bioprospection, Darcy Ribeiro Campus, University of Brasilia, 70910-900 Brasilia, DF, Brazil

7 Kiel University, Christian-Albrechts-Platz 4, 24118 Kiel, Germany

* Corresponding author. E-mail address: scicek@pharmazie.uni-kiel.de.

List of Contents

Table S1: ^{13}C NMR data of compounds **2**–**5**

Table S2: ^{1}H NMR data of compounds **2**–**5**

Table S3: Cytotoxic effects of compounds **1**–**5**

Table S4: Antifungal effects of compounds **1**–**5**

Table S5: Antibacterial effects of compounds **1**–**5**

Figure S1: HRESIMS spectrum of compound **1**

Figure S2: ^{1}H NMR spectrum of compound **1**

Figure S3: ^{13}C NMR spectrum of compound **1**

Figure S4: H,H COSY spectrum of compound **1**

Figure S5: HSQC spectrum of compound **1**

Figure S6: HMBC spectrum of compound **1**

Figure S7: NOESY spectrum of compound **1**

Table S1

^{13}C NMR data (150.90 MHz) of compounds **2–5** (δ in ppm)

Position	**2**[a]	**3**[b]	**4**[a]	**5**[a]
1	39.0	40.6	39.9	39.9
2	19.6	20.0	19.5	19.6
3	38.1	38.9	37.9	37.9
4	44.0	44.3	44.0	44.1
5	56.2	56.5	56.5	56.5
6	24.1	23.9	23.2	23.4
7	32.0	31.8	31.9	32.0
8	41.7	41.9	35.8	35.9
9	53.5	45.2	45.2	45.2
10	37.5	38.2	38.1	38.1
11	21.7	40.2	22.6	22.6
12	26.8	107.3	149.8	149.8
13	128.7	175.2	122.4	122.4
14	137.0	38.7	31.6	31.6
15	135.7	113.1	109.7	109.7
16	110.8	172.8	140.5	140.5
17	16.0	12.9	17.7	17.7
18	28.9	29.6	29.2	29.1
19	184.0	181.0	184.1	178.1
20	12.7	13.6	13.5	13.4
OCH_3				51.4

a) Values were recorded in chloroform-d_1. b) Values were recorded in a mixture (1:1) of chloroform-d_1 and methanol-d_4.

Table S2

^{1}H NMR data (400.33 MHz) of compounds **2**, **3**, **4**, and **5** (δ in ppm, J in Hz).

Position	**2**[a]	**3**[b]	**4**[a]	**5**[a]
1	0.95 m, 1.86 m	1.05 m, 1.77 m	1.06 m, 1.80 m	1.05 (m), 1.77 (m)
2	1.50 br d (13.9), 1.89 (m)	1.46 m, 1.81 m	1.49 m, 1.86 m	1.49 (m), 1.84 (m)
3	1.04 m, 2.17 br d (12.1)	1.01 m, 2.14 br d (12.7)	1.06 m, 2.20 br d (12.8)	1.05 (m), 2.20 (br d, J=16.9 Hz)
4				
5	1.13 dd (12.1, 3.6)	1.11 m	1.16 dd (12.1, 2.1)	1.13 (dd, J=14.3, 1.9 Hz)
6	1.94 m (2H)	1.73 m, 1.92 m	1.79 m, 1.95 dq (13.7, 2.2)	1.69 (m), 1.95 (dq)
7	0.86 m, 2.23 dq (12.6, 3.2)	1.33 m, 1.59 m	1.33 dq (9.3, 12.8), 1.75 m	1.32 (dq, J= 9.6, 13.1 Hz), 1.75 (m)
8	2.02 m	1.58 m	1.75 m	1.74 (m)
9	0.89 m	1.46 m	1.48 m	1.46 (m)
10				
11	1.07 dt (13.6, 4.8), 1.84 m	1.25 m, 2.40 dd (3.0, 12.9)	2.36 dd (10.4, 16.6), 2.56 m	2.38 dd (10.4, 16.5), 2.57 m
12	2.00 m, 2.33 br d (13.8)			
13				
14		2.92 m	2.63 m	2.62 m
15	6.80 dd (17.3, 11.0)	5.63 s	6.18 d (1.6)	6.17 d (1.7)
16	4.96 d (11.4), 5.11 d (17.1)		7.22 d (1.8)	7.21 d (1.7)
17	1.75 s	1.13 d (7.9)	0.98 d (7.0)	0.97 d (7.0)
18	1.23 s	1.19 s	1.28 s	1.21 s
20	0.76 s	0.71 s	0.82 s	0.71 s
21				3.65 s

a) Values were recorded in chloroform-d_1. b) Values were recorded in a mixture (1:1) of chloroform-d_1 and methanol-d_4.

Table S3

Cytotoxic effects of compounds **1**–**5** at a concentration of 100 µM. Effects are given in % growth inhibition. Positive control was doxorubicin.

	HaCaT	A-375	A-549	HCT-116	MB-231
Compound **1**	-	-	-	-	-
Compound **2**	-	38	-	26	-
Compound **3**	-	-	-	-	-
Compound **4**	-	-	-	-	-
Compound **5**	60	62	-	31	-
0.5% DMSO	-	-	-	-	-
Positive control	81	93	92	86	92

Table S4

Antifungal effects of compounds **1**–**5** at a concentration of 100 µM. Effects are given in % growth inhibition. Positive controls were nystatin (*C. albicans*), amphotericin B (*C. neoformans*), and clotrimazol (*T. rubrum*).

	C. albicans	*C. neoformans*	*T. rubrum*
Compound **1**	-	35	-
Compound **2**	-	-	-
Compound **3**	-	41	-
Compound **4**	-	-	-
Compound **5**	-	-	-
0.5% DMSO	-	-	-
Positive control	93	100	99

Table S5

Antibacterial effects of compounds **1**–**5** at a concentration of 100 µM. Effects are given in % growth inhibition. Positive controls were chloramphenicol (MRSA, *K. pneumoniae*, *E. coli*), ampicillin (*E. faecium*), doxycylcline (*A. baumanii*), and polymyxin B (*P. aeruginosa*).

	MRSA	*E. faecium*	*K. pneumoniae*	*A. baumannii*	*P. aeruginosa*	*E. coli*
Compound **1**	31	-	-	-	-	-
Compound **2**	80	-	-	-	-	-
Compound **3**	-	-	-	-	-	-
Compound **4**	98	92	-	-	-	-
Compound **5**	-	-	-	-	-	21
0.5% DMSO	-	-	-	-	-	-
Positive control	100	100	99	100	99	97

Fig. S1:

HRESIMS spectrum of compound **1**

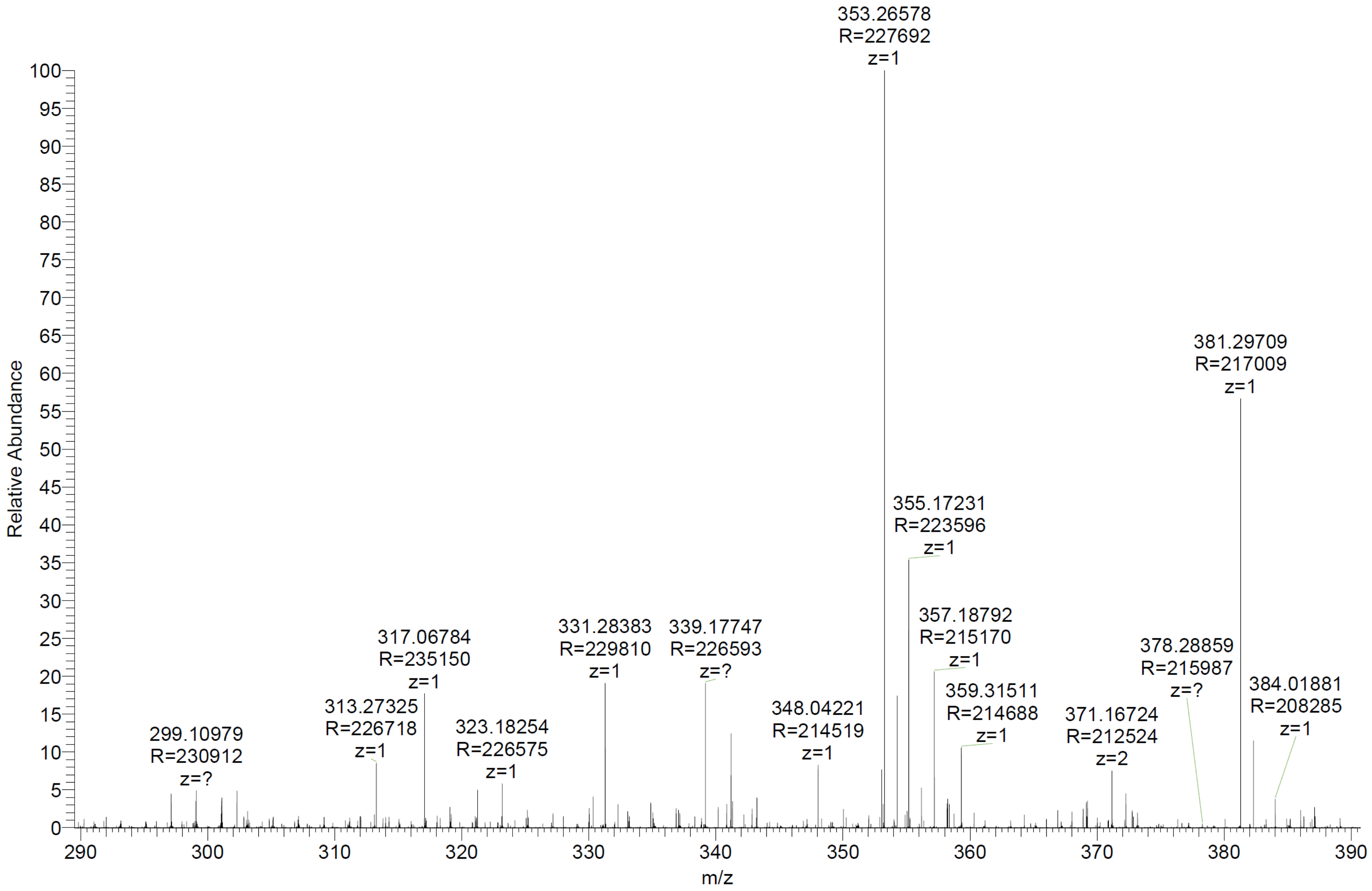

Fig. S2:

^{1}H NMR spectrum (600.13 MHz) of compound **1** measured in chloroform-d_1

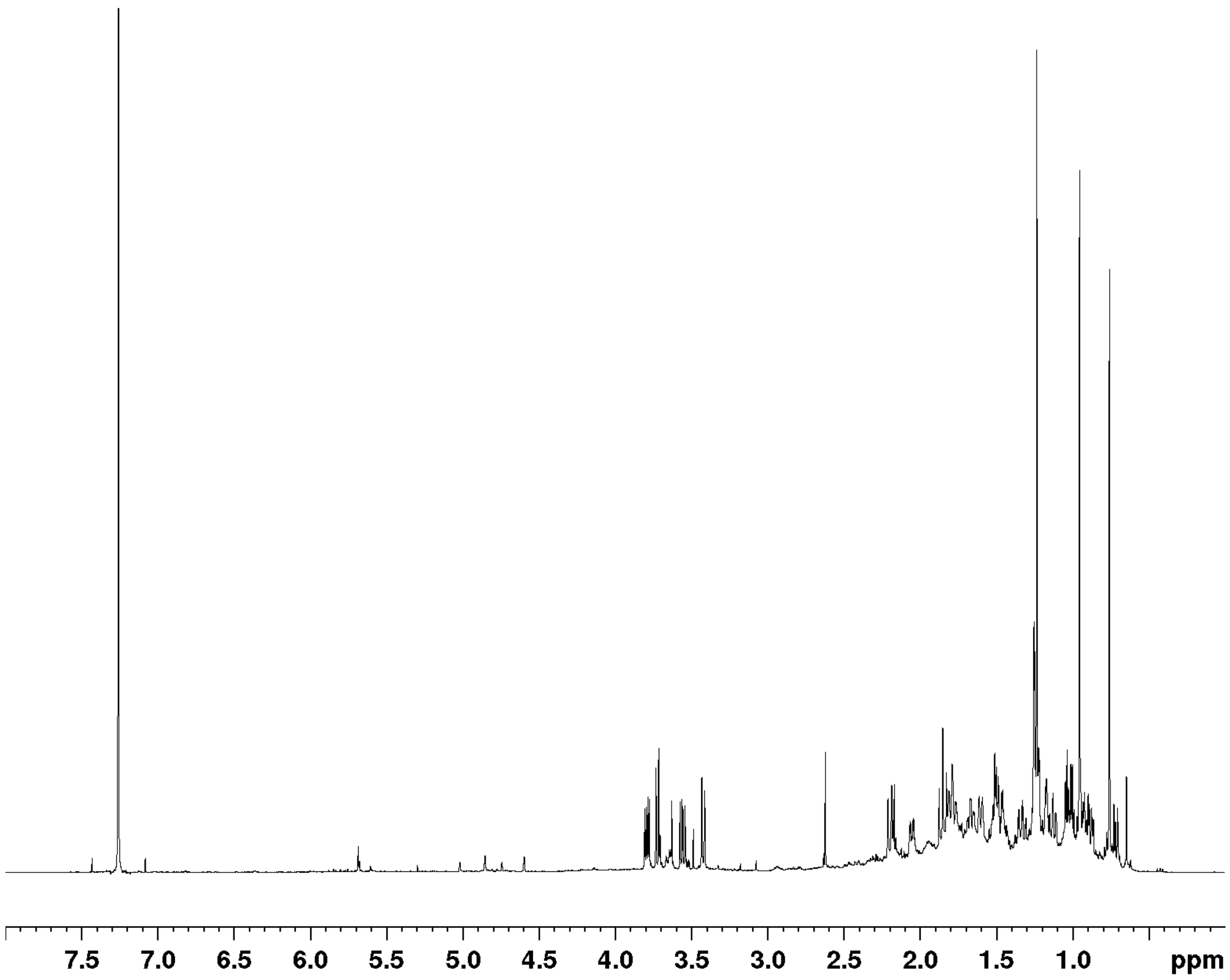

Fig. S3:

^{13}C NMR (150.90 MHz) spectrum of compound **1** measured in chloroform-d_1

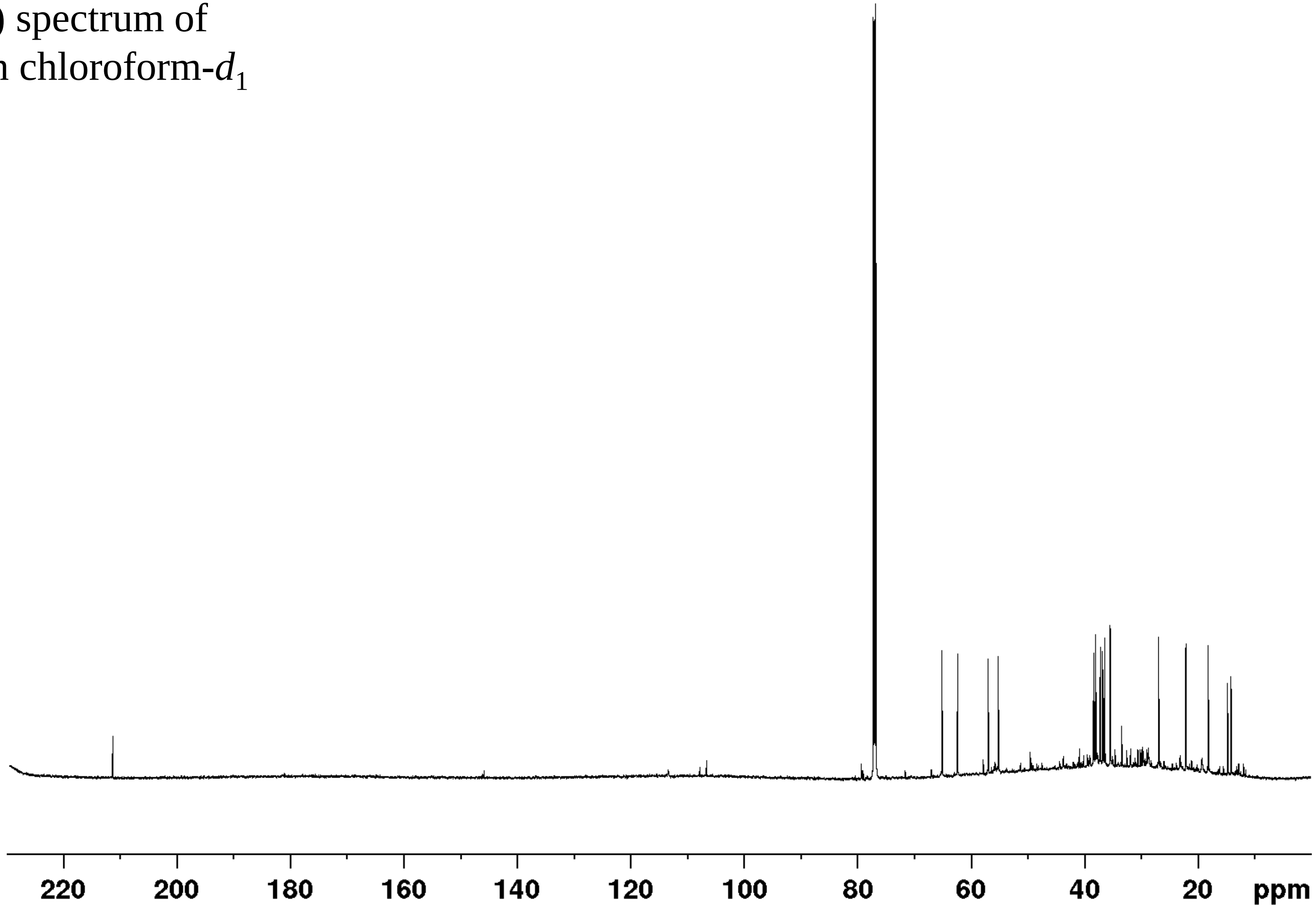

Fig. S4:

H,H COSY spectrum of compound **1** measured in chloroform-d_1

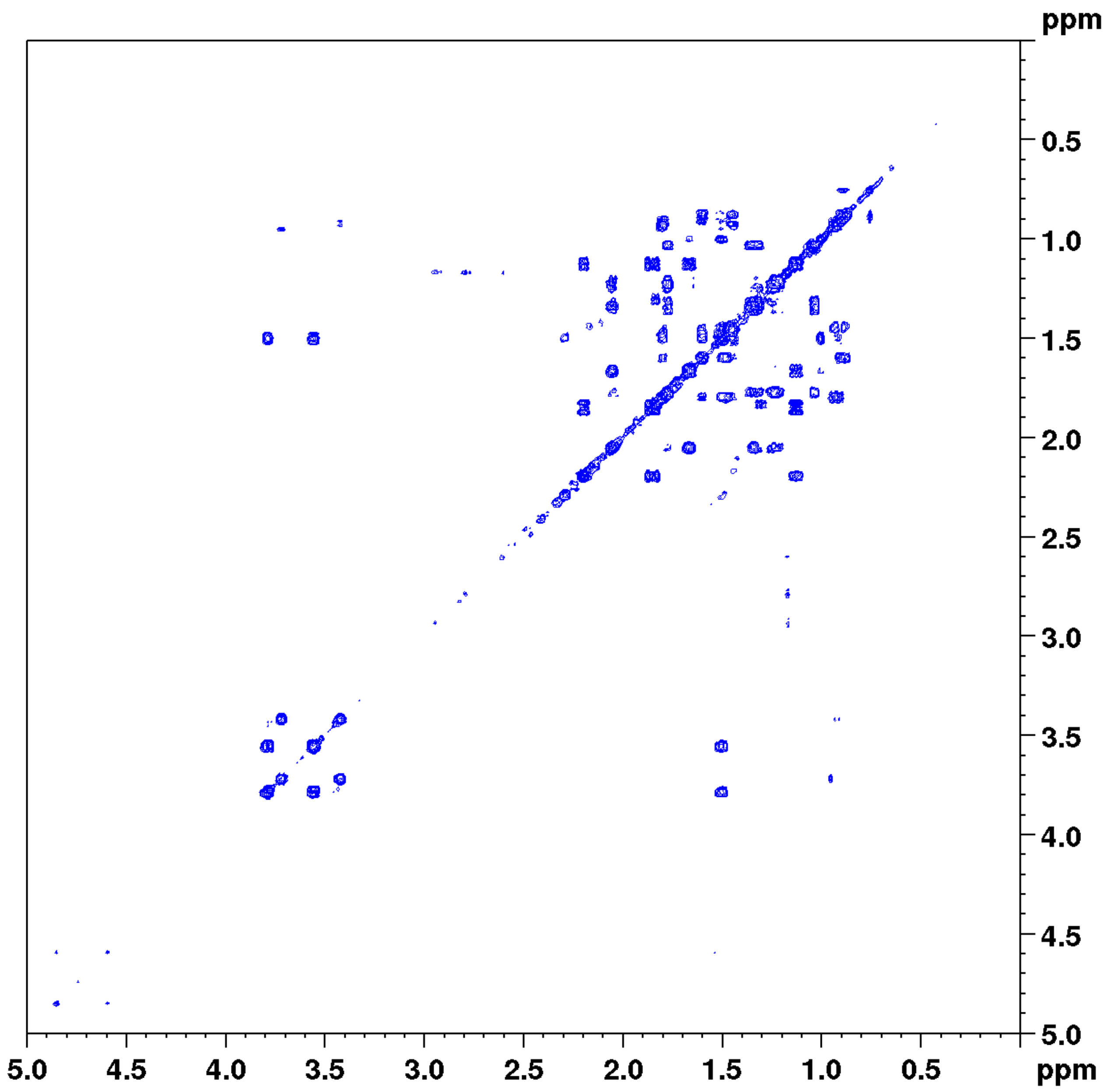

Fig. S5:

HSQC spectrum of compound **1** measured in chloroform-d_1

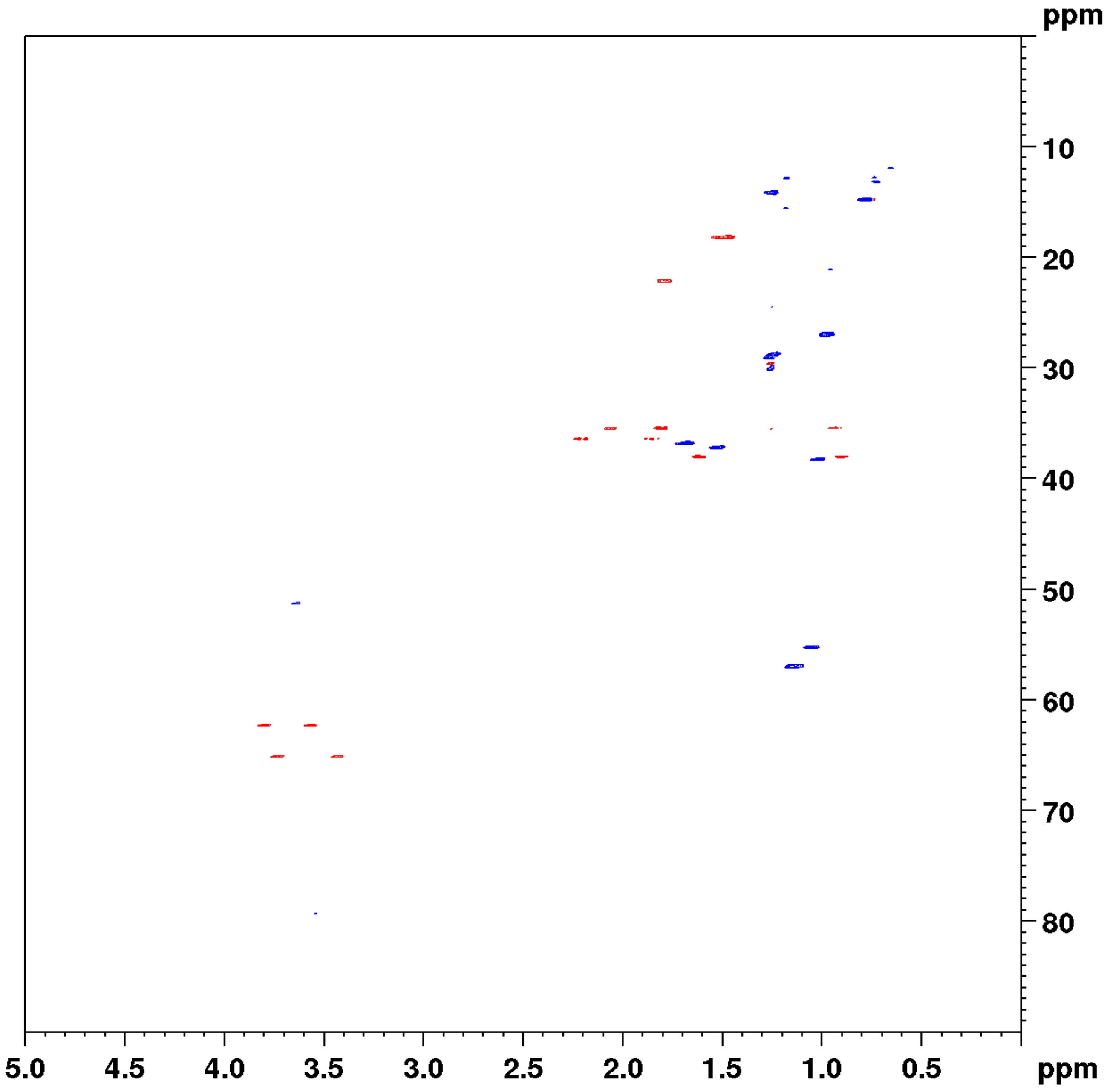

Fig. S6:

HMBC spectrum of compound **1** measured in chloroform-d_1

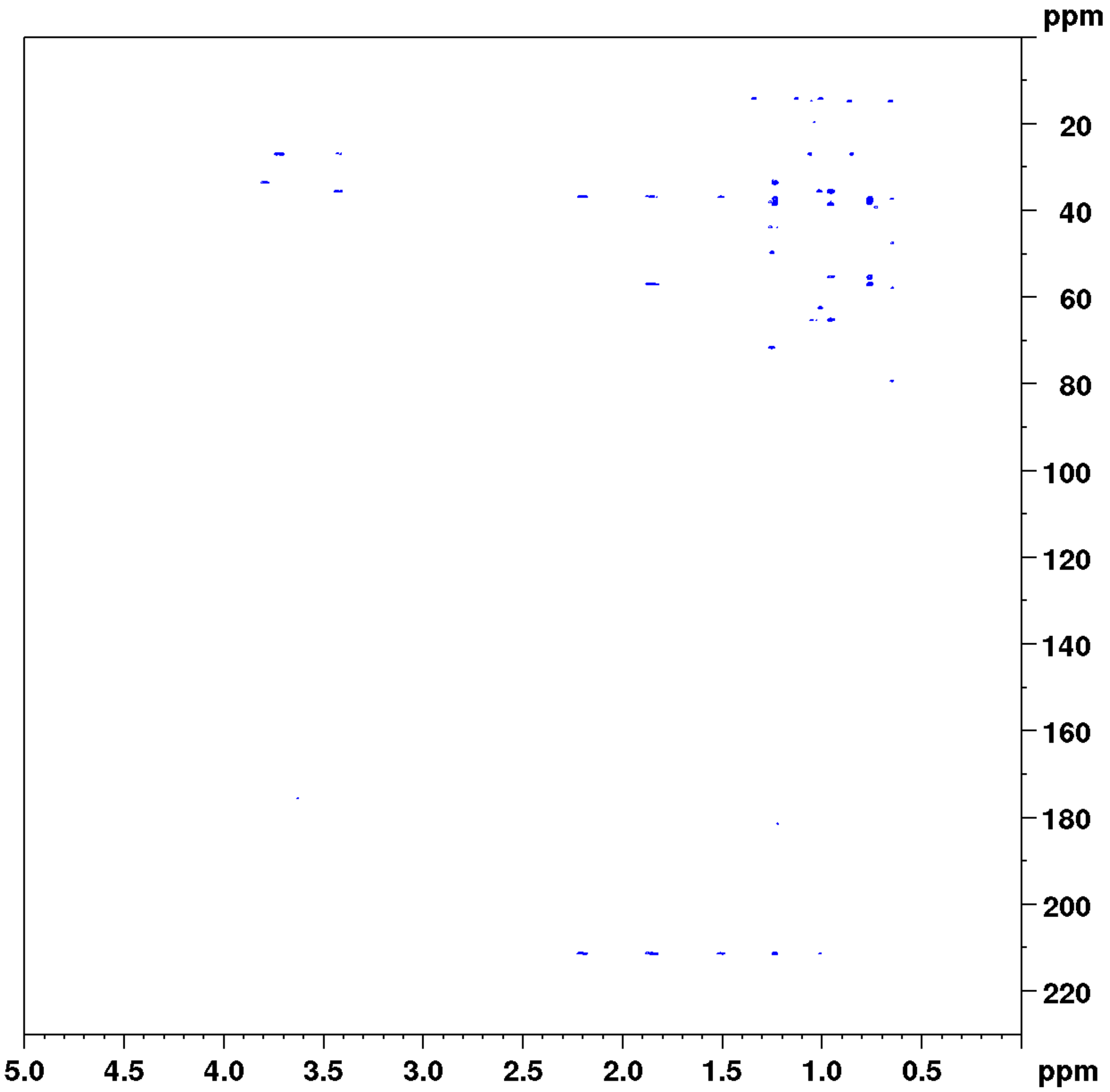

Fig. S7:

NOESY spectrum of compound **1** measured in chloroform-d_1

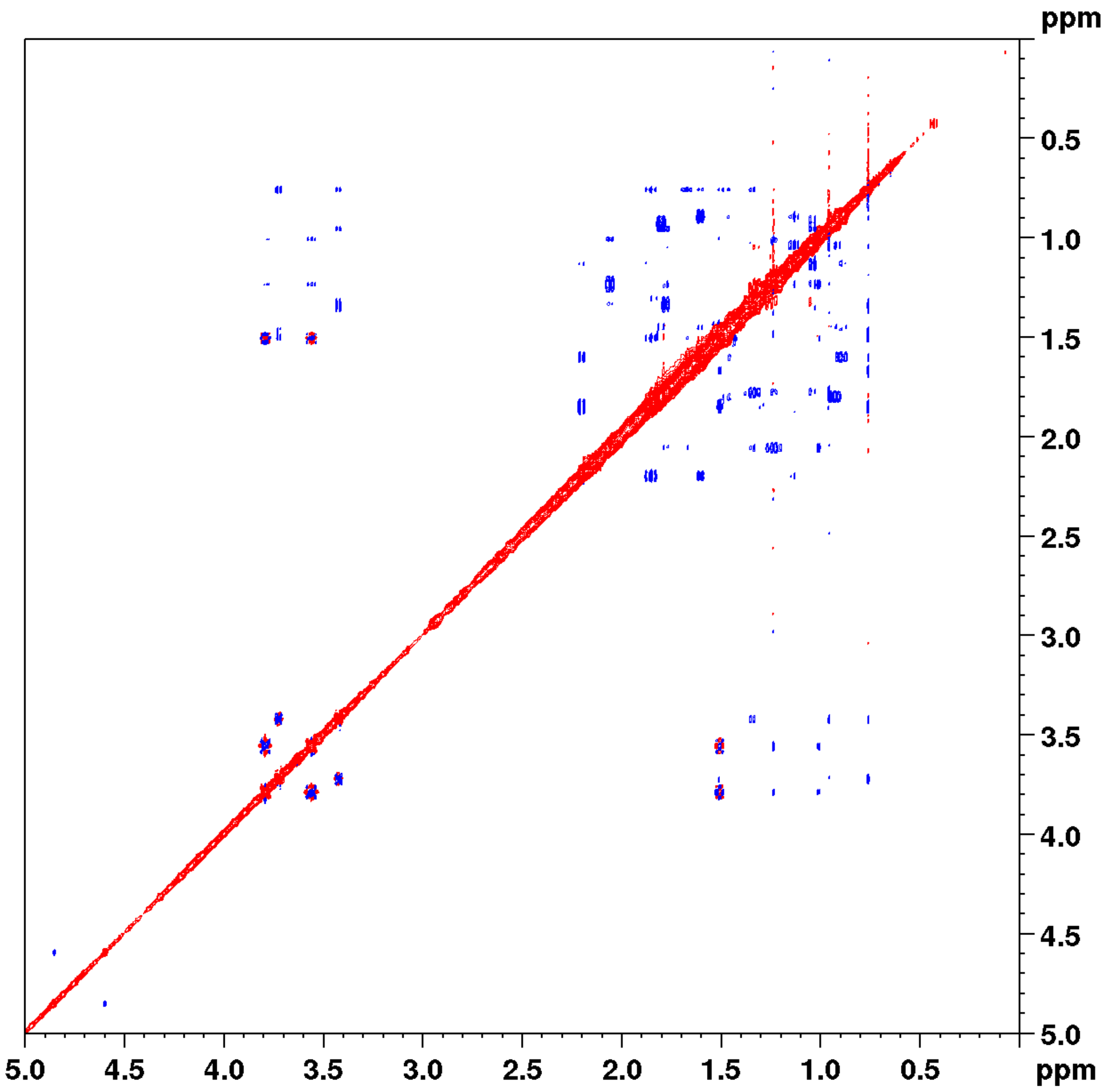

Supplement: Supplementary file 1 [file plants-12-00099-s001.zip › plants-2078245-supplementary.pdf]
